# Synaptic remodeling follows upper motor neuron hyperexcitability in a rodent model of TDP-43

**DOI:** 10.3389/fncel.2023.1274979

**Published:** 2023-10-24

**Authors:** Marcus S. Dyer, G. Lorenzo Odierna, Rosemary M. Clark, Adele Woodhouse, Catherine A. Blizzard

**Affiliations:** ^1^Menzies Institute for Medical Research, College of Health and Medicine, University of Tasmania, Hobart, TAS, Australia; ^2^Department of Pharmaceutical and Pharmacological Sciences, Center for Neurosciences, Vrije Universiteit Brussel, Brussels, Belgium; ^3^Tasmanian School of Medicine, College of Health and Medicine, University of Tasmania, Hobart, TAS, Australia; ^4^Wicking Dementia Research and Education Centre, College of Health and Medicine, University of Tasmania, Hobart, TAS, Australia

**Keywords:** amyotrophic lateral sclerosis, motor neuron disease, TDP-43, hyperexcitability, excitatory postsynaptic currents, synapse, timeline

## Abstract

Amyotrophic Lateral Sclerosis (ALS) is an incurable disease characterized by relentlessly progressive degeneration of the corticomotor system. Cortical hyperexcitability has been identified as an early pre-symptomatic biomarker of ALS. This suggests that hyperexcitability occurs upstream in the ALS pathological cascade and may even be part of the mechanism that drives development of symptoms or loss of motor neurons in the spinal cord. However, many studies also indicate a loss to the synaptic machinery that mediates synaptic input which raises the question of which is the driver of disease, and which is a homeostatic response. Herein, we used an inducible mouse model of TDP-43 mediated ALS that permits for the construction of detailed phenotypic timelines. Our work comprehensively describes the relationship between intrinsic hyperexcitability and altered synaptic input onto motor cortical layer 5 pyramidal neurons over time. As a result, we have constructed the most complete timeline of electrophysiological changes following induction of TDP-43 dysfunction in the motor cortex. We report that intrinsic hyperexcitability of layer 5 pyramidal neurons precedes changes to excitatory synaptic connections, which manifest as an overall loss of inputs onto layer 5 pyramidal neurons. This finding highlights the importance of hyperexcitability as a primary mechanism of ALS and re-contextualizes synaptic changes as possibly representing secondary adaptive responses. Recognition of the relationship between intrinsic hyperexcitability and reduced excitatory synaptic input has important implications for the development of useful therapies against ALS. Novel strategies will need to be developed that target neuronal output by managing excitability against synapses separately.

## 1. Introduction

Amyotrophic lateral sclerosis (ALS) is an incurable neurodegenerative disease characterized by progressive loss of voluntary muscle function. Symptoms of ALS indicate dysfunction of upper and lower motor neurons and can include muscle spasticity, rigidity and paralysis. One of the most specific biomarkers for ALS is cortical hyperexcitability, measurable via diagnostic transcranial magnetic stimulation assays (Vucic and Kiernan, 2008; Vucic et al., 2011, 2021). Clinical studies of patients have identified that individuals with a familial history of ALS develop cortical hyperexcitability before presentation of symptoms (Vucic et al., 2008). This suggests that hyperexcitability occurs upstream in the pathological cascade and may even be part of the mechanism that drives development of symptoms or loss of motor neurons in the spinal cord. This has never been formally demonstrated, however, and it remains possible that cortical hyperexcitability occurs as part of an adaptive mechanism to a yet identified more primary insult. Differentiating between primary pathophysiology and secondary plastic/homeostatic adaptations in ALS is an extremely challenging but necessary exercise in the development of useful therapeutics for patients. This is because targeting adaptive mechanisms is not likely to produce meaningful outcomes for patients and may even worsen symptoms.

Rodent models of ALS have provided valuable mechanistic information pertaining to the cortical hyperexcitability phenomenon. Multiple studies from independent research groups have demonstrated that layer V pyramidal neurons (LVPNs) in the rodent motor cortex are often intrinsically hyperexcitable (Saba et al., 2016; Zhang et al., 2016; Kim et al., 2017; Dyer et al., 2021a). This suggests that cortical (network) hyperexcitability may be explained, in part, by increased excitability of LVPNs, some of which are directly responsible for transmitting signals from the cortex to the spinal cord. Due to the ability to resolve the early stages of the disease progression, rodent models have also provided insights into the evolution of neurophysiological changes in LVPNs. In the SOD1^G93A^ mouse model of ALS, LVPNs demonstrate increased intrinsic excitability as early as postnatal day (P)5 (Kim et al., 2017). This increased excitability is present at pre-and post-symptomatic stages in adult mice (Saba et al., 2016; Kim et al., 2017), indicating a relative stability of the phenotype over long periods of time. Increased excitability of LVPNs has also been observed in the TDP-43^A315T^ mouse model (Zhang et al., 2016) which suggests that hyperexcitability is not a specific outcome of individual genetic mutations but rather is a common phenotype of ALS-related molecular insults. Observations of increased neuronal activity in FUS (Scekic-Zahirovic et al., 2021) and C9orf72 (Amalyan et al., 2022) *in vivo* mouse models of ALS support this idea, as do studies *in vitro* that report increased excitability of rodent primary neurons (Pieri et al., 2003; Kuo et al., 2005; Pieri et al., 2009) and human induced pluripotent stem cell-derived motor neurons (Wainger et al., 2014; Devlin et al., 2015).

Results from investigations into synaptic inputs onto LVPNs have provided a less consistent picture, albeit one that agrees with the general notion of altered neurophysiology preceding onset of symptoms. Fogarty et al. (2015) found that the frequency of spontaneous excitatory postsynaptic currents (sEPSCs) onto LVPNs in SOD1^G93A^ mice was increased as early as P21 despite a decrease in the density of dendritic spines and regression of apical dendrites at P28. Saba et al. (2016) also reported that the frequency of sESPCs was increased in the SOD1^G93A^ mouse model at a similar time point (P26-31) but instead found that LVPN dendritic morphology was expanded rather than reduced. In the TDP-43^A315T^ mouse model, dendritic morphology and mEPSC frequency appear normal at P20-22 and P30 (Zhang et al., 2016; Handley et al., 2017) but then are both decreased at P60, before loss of LVPNs at P90 (Handley et al., 2017). Contradicting this, sEPSC frequency is increased between P26-35 in the TDP-43^Q331K^ mouse model, as are the number of dendritic spines on LVPNs (Fogarty et al., 2016a). Although EPSCs have not been measured in neonatal mice for any mouse models of ALS, morphological dendrite assessments in the SOD1^G93A^ model indicate loss of spines as early as P8-15 (Fogarty et al., 2016b), suggesting that synaptic changes may occur as early as those observed for intrinsic hyperexcitability.

To date, no study has been able to comprehensively describe the relationship between intrinsic hyperexcitability and altered synaptic input onto LVPNs over time. This is a critical missing piece of the puzzle because we still do not know which of the phenotypes in ALS are a consequence of primary/causative molecular processes and which are homeostatic adaptations to these primary insults. As such, the core question remains: do synaptic changes precede intrinsic LVPN hyperexcitability or vice versa? Answering this question is critical for the development of useful therapies for ALS, which need to take primary and secondary neurophysiological changes into account.

## 2. An inducible model of ALS to build a timeline of neurophysiological changes

We have recently been working to decipher how neurophysiological changes in ALS relate to each other by taking advantage of a tetracycline-inducible binary expression transgenic system. The system allows for the generation of transgenic mice that express a nuclear localization sequence (NLS)-deficient human TDP-43 in LVPNs in the motor cortex (Dyer et al., 2021a) and not lower motor neurons in the spinal cord (Reale et al., 2023) using the CaMKIIa promoter (hereon referred to as the TDP-43^ΔNLS^ mouse line). Severe overexpression of TDP-43^ΔNLS^ causes neurodegeneration and molecular signatures associated with ALS (Igaz et al., 2011), mimicking the cytoplasmic mis-localization of TDP-43 identified as a pathological hallmark of motor neurons in 97% of all ALS cases (Mackenzie et al., 2007; Maekawa et al., 2009). Because expression of TDP-43^ΔNLS^ is enriched in the motor cortex but otherwise excluded in lower motor neurons, phenotypes can be tentatively ascribed as being region-specific. Within the brain, expression is enriched in the cortex where the CaMKIIa promoter is active in both inhibitory and excitatory neurons, including LVPNs (Schönig et al., 2012; Dyer et al., 2021a; Veres et al., 2023). It must be noted that astrocytes and other support cells do not express CaMKIIa (Vallano et al., 2000), and as such the model does not interrogate the consequences of overexpressing TDP-43 in these cells. The main advantage of the system is that it permits for high temporal control of transgene expression such that strict timelines can be constructed to interrogate changes following initiation of mislocalization. This is particularly important for understanding the pathogenesis of ALS since assessment of pTDP-43 pathology in sporadic and familial ALS patients indicates that mislocalization of TDP-43 occurs in discrete stages (post-developmentally) as the disease progresses (Braak et al., 2013; Brettschneider et al., 2013).

Initiating expression at P30, followed by 30 days of expression (P30 + 30) in the TDP-43^ΔNLS^ mouse line results in intrinsic hyperexcitability of LVPNs (Dyer et al., 2021a). Whole-cell patch-clamp of LVPNs at earlier time points has revealed that the increased excitability is detectable at P30 + 20 and not P30 + 10 (Reale et al., 2023). Because of the restricted expression pattern permitted by CaMKIIa, this hyperexcitability can be attributed to processes specific to the cortex. Despite this, it remains unclear whether it is the result of mechanisms autonomous to LVPNs or whether it originates from neurophysiological changes in other cortical neurons (for example, from adaptive changes to the neurophysiology of cortico-cortical neurons, or local inhibitory interneurons). At P30 + 30, sEPSCs on LVPNs have decreased frequency and mEPSCs have decreased amplitude, suggesting that by this time point synaptic changes have taken place in addition to the intrinsic hyperexcitability. As of yet, it remains unknown when synaptic changes begin in this mouse line, since they have only been explored at P30 + 30.

## 3. Synaptic changes occur after the induction of intrinsic hyperexcitability

**Figure 1:**
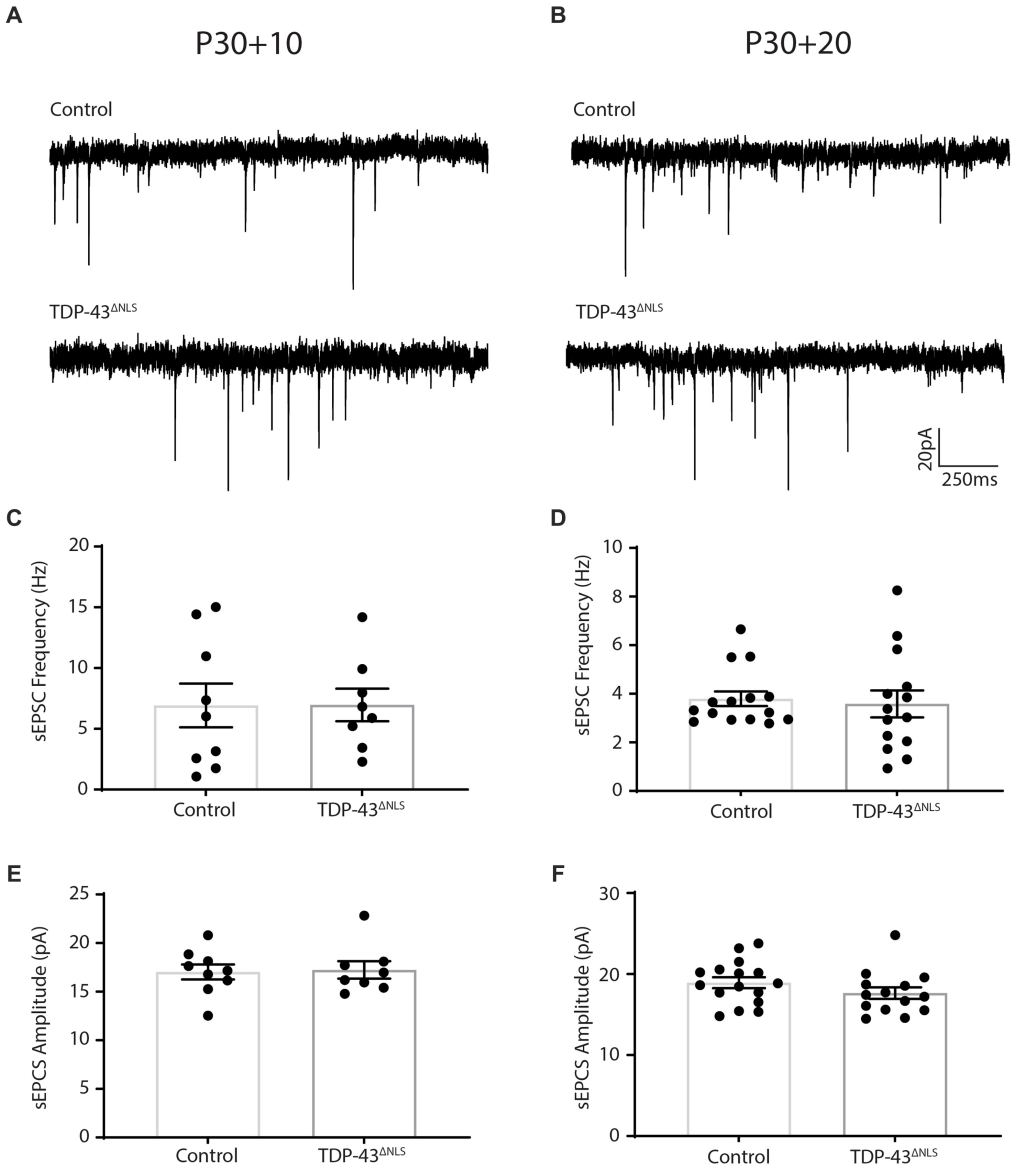
Spontaneous excitatory postsynaptic current frequency and amplitude is unchanged following 10 and 20 days of TDP-43^ΔNLS^ expression in layer 5 pyramidal neurons. **(A,B)** Representative traces of spontaneous excitatory postsynaptic currents (sEPSCs) from CaMKIIa-tTA/+ and +/+ mice (Control) and mice expressing a nuclear localization sequence-deficient human TDP-43 (TDP-43^ΔNLS^) following 10 **(A)** and 20 **(B)** days of transgenic expression. **(C,D)** The frequency of sEPSCs is not different between control and TDP-43^ΔNLS^ mice at 10 days post expression (**C**; P30 + 10) and 20 days post expression (**D**; P30 + 20). **(E,F)** The amplitude of sEPSCs is not different between control and TDP-43^ΔNLS^ mice at 10 days post expression **(E)** and 20 days post expression **(F)**. Experiments conducted in accordance with the Australian Code of Practice for the Care and Use of Animals for Scientific Purposes and approved by the Animal Ethics Committee of the University of Tasmania (A16593). Mice were group housed (2–5 per cage) in individually ventilated cages on a 12-h light–dark cycle. Female mice were excluded from this study. CaMKIIa-tTa (The Jackson Laboratory Stock # 003010) and tetO-TDP-43^ΔNLS^ (The Jackson Laboratory Stock# 014650) were fully backcrossed to a C57Bl/6J background for a minimum of 10 generations (confirmed test of 99.8–100% C57Bl/6J background). To collect tissue for electrophysiological assessments, mice were sacrificed via intraperitoneal injection of with sodium pentobarbital (200 mg/kg) followed by trans-cardial perfusion with ice cold carbogenated (95% O_2_, 5% CO_2_) perfusion solution containing (in mM): 92 choline chloride (Sigma-Aldrich Cat# C7527), 2.5 KCl (Sigma-Aldrich Cat# S9541), 1.2 NaH_2_PO4 (Sigma-Aldrich Cat# S5011), 30 NaHCO_3_ (Sigma-Aldrich Cat# S5761), 20 HEPES (Sigma-Aldrich Cat# H3784), 25 Glucose (Sigma-Aldrich Cat# G7021), 0.5 CaCl_2_ (Sigma-Aldrich Cat# C3306), 10 MgSO_4_•7H_2_O (Sigma-Aldrich Cat# M2773), 5 sodium ascorbate (Sigma-Aldrich Cat# A4032), 2 Thiourea (Sigma-Aldrich Cat# T8656), 3 sodium pyruvate (Sigma-Aldrich Cat# P5280), 5 N-Acetyl-L-Cysteine (Sigma-Aldrich Cat# A9165). Brains were immediately sliced (coronal sections) at 400 μm on a Leica VT1200s vibratome. Acute slices were recovered in a holding solution containing (in mM): 92 NaCl, 2.5 KCl, 1.2 NaH_2_PO_4_, 30 NaHCO_3_, 20 HEPES, 2 CaCl_2_, 2 MgCl_2_ (Sigma-Aldrich Cat# M2392) and 25 Glucose. Borosilicate glass capillaries (Harvard Apparatus) were pulled to produce patch pipettes with a tip resistance of 3–5 MΩ (P1000 pipette puller, Sutter Instruments). The intracellular solution contained (in mM): 120 Cs-methanesulfunate (Sigma-Aldrich Cat# C1426), 20 CsCl; (Sigma Aldrich Cat# C4036), 10 HEPES, 10 EGTA (Sigma-Aldrich Cat# O3777), 2 MgCl_2_, 2 Na_2_ATP (Abcam Cat# ab120385) and 0.3 NaGTP (Abcam Cat# ab146528) and CsOH (Sigma Aldrich Cat# 232041), at pH 7.4 and 280–290 mOsm. Slices were continuously perfused (> 4 mL/min) using aCSF (warmed to 30°C) containing 100 μM picrotoxin (Abcam Cat# ab120315) to eliminate GABAergic currents. The motor cortex was identified using a brain atlas at low magnification and layer V was identified using a 40x objective based on the presence of very large, sparse pyramidal neurons. Whole-cell patch-clamp recordings of primary motor cortex layer V pyramidal neurons (uncompensated series resistance of <20 MΩ) were made using a Heka EPC10 USB amplifier via the Heka Elektronik Patchmaster software. Voltage clamp recordings of EPCSs were made at-70 mV for 2 min, were sampled at 20 kHz and filtered with a low pass filter at 3 kHz. All recordings had a series resistance <15 MΩ and noise <10 pA, only cells with a stable series resistance over the course of the 2 min recording were used. Blinding was carried out for electrophysiological experiments and analysis. Each data point represents an individual cell. Groups were compared using a student’s t-test with significance threshold set to *p* < 0.05.

**Figure 2:**
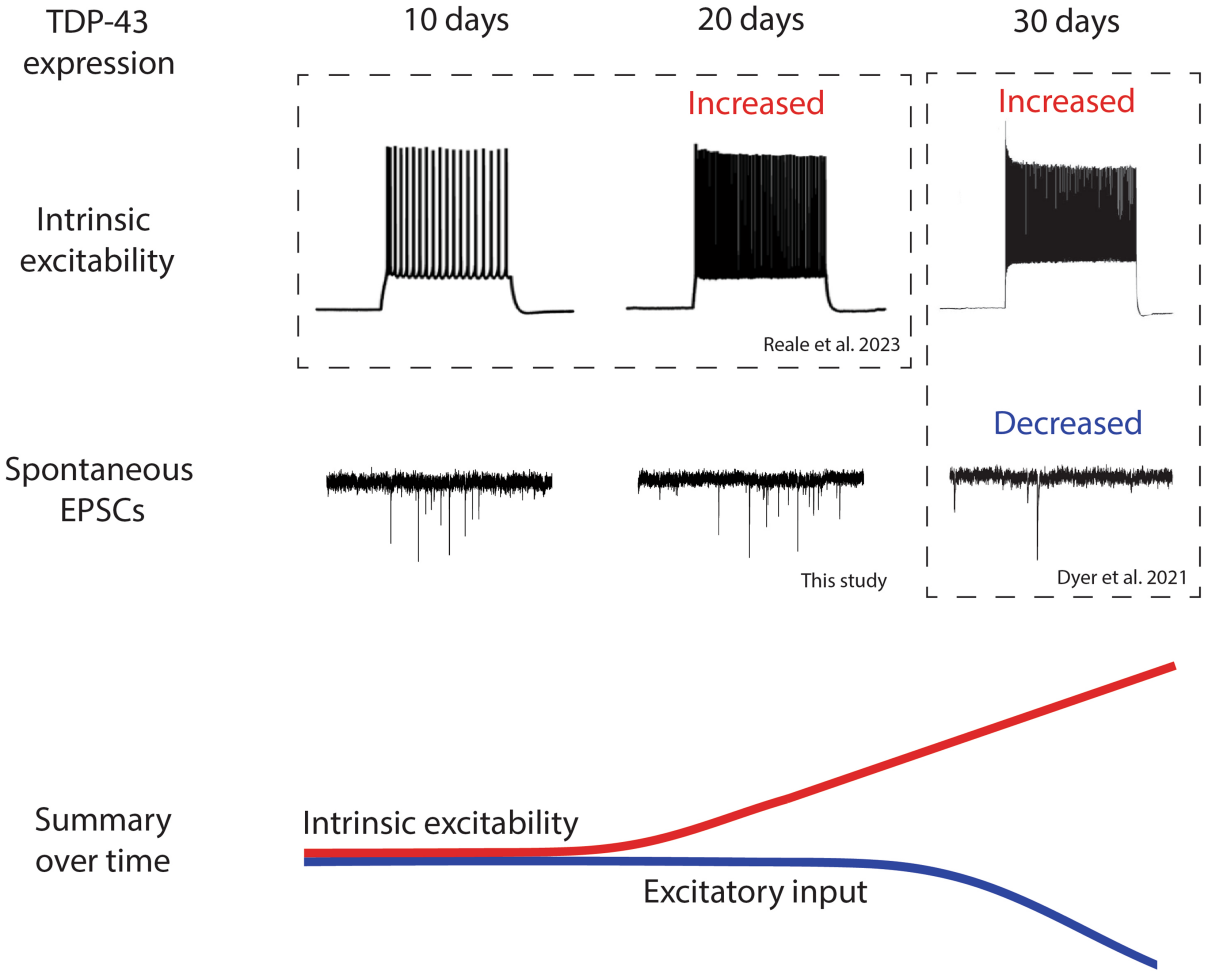
Timeline of electrophysical changes in layer 5 pyramidal neurons following TDP-43^ΔNLS^ expression shows that intrinsic hyperexcitability precedes reduced excitatory input. A summary of the electrophysiological findings in layer 5 pyramidal neurons from the TDP-43^ΔNLS^ mouse line. Intrinsic excitability and excitatory input are not different at 10 days post expression of TDP-43. After 20 days of TDP-43 expression, layer 5 pyramidal neurons become intrinsically hyperexcitable but excitatory input does not change. After 30 days of expression, intrinsic hyperexcitability continues to increase and excitatory input decreases. Changes are summarized by colored lines over time (red represents intrinsic excitability, blue represents excitatory input). Traces adapted from Dyer et al. (2021a) and Reale et al. (2023) indicated by dotted boxes.

To expand our exploration of the relationship between synaptic input onto LVPNs and their intrinsic excitability, we performed whole-cell patch-clamp recordings of sEPSCs from LVPNs in the TDP-43^ΔNLS^ mouse line at two as yet unexplored time points; 10-and 20-days post initiation of expression (P30 + 10 and P30 + 20, respectively). We quantified the frequency and amplitude of sEPSCs and found that at P30 + 10, control and TDP-43^ΔNLS^ LVPNs could not be statistically differentiated (Figure 1). Our previous observations indicated that at P30 + 10, there was no difference in intrinsic excitability between control and TDP-43^ΔNLS^ (Reale et al., 2023). As such, after 10 days of TDP-43^ΔNLS^ expression, intrinsic excitability and excitatory synaptic input onto LVPNs are both unchanged. We next measured spontaneous events at P30 + 20 and found that there, again, was no statistically significant difference in both the frequency and amplitude of sEPSCs (Figure 2). Contrary to the P30 + 10 time point, we have previously demonstrated that LVPNs are hyperexcitable at P30 + 20 (Reale et al., 2023) and that this persists at P30 + 30 (Dyer et al., 2021a). Thus, in the TDP-43^ΔNLS^ mouse model, intrinsic excitability precedes synaptic changes in LVPNs. Lastly, we examined inhibitory postsynaptic currents (IPSCs) from LVPNs at P30 + 30 to test whether the synaptic effects were specific to excitatory connections. Measurements of mIPSC and sIPSC amplitude and frequency from control and TDP-43^ΔNLS^ LVPNs revealed no statistically significant differences (Supplementary Figure S1).

The observation of hyperexcitability preceding changes to sEPSCs in LVPNs highlights the possibility that synaptic changes occur as an intrinsic homeostatic adaptation to increased neuronal firing. Although we have not measured the firing output of LVPNs in the TDP-43^ΔNLS^ mouse model, the changes are consistent with what is known about synaptic scaling (Turrigiano, 2008). Inducing increased activity of neurons is known to suppress EPSC amplitude, and by P30 + 30 both the frequency and amplitude of EPSCs have both decreased in LVPNs (Dyer et al., 2021a). The decreased frequency of EPSCs in this mouse model are attributed to decreased dendritic spine density (Dyer et al., 2021b), which rules out alterations to spontaneous neurotransmitter release as an explanation. This decrease occurred across the entire dendritic arbor of LVPNs, indicating that loss of synaptic input is driven by mechanisms specific to LVPNs (such as their activity) rather than originating from any discrete subpopulation of excitatory neurons presynaptic to LVPNs. Classical synaptic downscaling has been posited to act as a mechanism that can eliminate dendritic spines by driving reductions of postsynaptic complexes to such an extent that they destabilize and are structurally lost.

For the first time, we have provided the most comprehensive timeline of events that specifically links intrinsic excitability and synaptic input changes in LVPNs in an individual mouse model of ALS. When combined, our data provides the following timeline narrative following TDP-43^ΔNLS^ expression in the cortex (Figure 2): (1) After 10 days of expression, LVPNs display no signs of neurophysiological change, (2) After 20 days of expression, LVPNs have become intrinsically hyperexcitable but there are no detectable changes in sEPSC amplitude or frequency, (3) After 30 days of expression, sEPSC frequency onto LVPNs is decreased and dendritic spines are lost. Despite this, LVPNs remain intrinsically hyperexcitable.

## 4. Discussion

Our findings clearly demonstrate that neurophysiological changes in ALS follow a dynamic pattern of evolving changes (Figure 2). This is not surprising given the inherent plasticity of neurons but is critically important to recognize in the process of developing therapies for ALS patients. Targeting enhanced intrinsic hyperexcitability or synaptic strength alone may not be helpful for patients because of how inherently interdependent both changes are to each other. Also, since changes in synapses follow the induction of intrinsic hyperexcitability they may represent a protective adaptation that normalizes neuronal output via a process such as synaptic scaling (Turrigiano, 2012). New therapies for ALS might therefore need to focus on managing synaptic transmission while specifically dampening intrinsic excitability of upper motor neurons. This could be achieved by (1) targeting mediators of synaptic homeostatic processes activated in ALS-like mice, such as Scnn1a (Orr et al., 2020), and (2) targeting ion channels implicated in LVPN hyperexcitability in ALS-like mice, such as Nav1.6, HCN or KCNQ channels (Buskila et al., 2019; Saba et al., 2019).

The idea that primary and secondary effects need to be considered when developing treatments for ALS extends beyond considerations of the excitability changes to individual motor neurons. It remains unclear, for example, how cortical hyperexcitability fits into the puzzle of ALS. Although hyperexcitability has been posited as a degeneration-inducing phenomenon, this has not yet been empirically verified. It remains possible that cortical hyperexcitability in ALS represents an adaptative network response to decreased motor output. Prolonged reductions in muscle activity can produce adaptations in the human cortical motor system, including increasing cortical motor excitability (Clark et al., 2008). This raises the possibility that cortical hyperexcitability may be a consequence of decreased motor output. If cortical hyperexcitability is required to maintain lower motor neuron activity and functional motor output, then efforts to suppress it may have unintended consequences, which will depend on the excitability state of the lower motor neuron and the stage of disease. Understanding how different neurophysiological changes fit together in ALS will be essential to develop appropriate therapeutic strategies to treat patients’ needs.

## Data availability statement

The raw data supporting the conclusions of this article will be made available by the authors, without undue reservation.

## Ethics statement

The animal study was approved by the University of Tasmania’s Animal Ethics Committee. The study was conducted in accordance with the local legislation and institutional requirements.

## Author contributions

MD: Conceptualization, Formal analysis, Investigation, Writing – review & editing. GO: Writing – original draft, Writing – review & editing. RC: Writing – review & editing. AW: Conceptualization, Methodology, Supervision, Writing – review & editing. CB: Conceptualization, Funding acquisition, Project administration, Resources, Supervision, Writing – review & editing.

## References

[ref1] Amalyan S. Tamboli S. Lazarevich I. Topolnik D. Bouman L. H. Topolnik L. (2022). Enhanced motor cortex output and disinhibition in asymptomatic female mice with C9orf72 genetic expansion. Cell Rep. 40:111043. doi: 10.1016/j.celrep.2022.111043, PMID: 35793625

[ref2] Braak H. Brettschneider J. Ludolph A. C. Lee V. M. Trojanowski J. Q. Del Tredici K. (2013). Amyotrophic lateral sclerosis—a model of corticofugal axonal spread. Nat. Rev. Neurol. 9, 708–714. doi: 10.1038/nrneurol.2013.221, PMID: 24217521 PMC3943211

[ref3] Brettschneider J. Del Tredici K. Toledo J. B. Robinson J. L. Irwin D. J. Grossman M.. (2013). Stages of ptdp-43 pathology in amyotrophic lateral sclerosis. Ann. Neurol. 74, 20–38. doi: 10.1002/ana.23937, PMID: 23686809 PMC3785076

[ref4] Buskila Y. Kékesi O. Bellot-Saez A. Seah W. Berg T. Trpceski M.. (2019). Dynamic interplay between H-current and M-current controls motoneuron hyperexcitability in amyotrophic lateral sclerosis. Cell Death Dis. 10:310. doi: 10.1038/s41419-019-1538-9, PMID: 30952836 PMC6450866

[ref5] Clark B. C. Issac L. C. Lane J. L. Damron L. A. Hoffman R. L. (2008). Neuromuscular plasticity during and following 3 wk of human forearm cast immobilization. J. Appl. Physiol. 105, 868–878. doi: 10.1152/japplphysiol.90530.2008, PMID: 18635877

[ref6] Devlin A. C. Burr K. Borooah S. Foster J. D. Cleary E. M. Geti I.. (2015). Human ipsc-derived motoneurons harbouring Tardbp or C9orf72 Als mutations are dysfunctional despite maintaining viability. Nat. Commun. 6:5999. doi: 10.1038/ncomms6999, PMID: 25580746 PMC4338554

[ref7] Dyer M. S. Reale L. A. Lewis K. E. Walker A. K. Dickson T. C. Woodhouse A.. (2021a). Mislocalisation of Tdp-43 to the cytoplasm causes cortical hyperexcitability and reduced excitatory neurotransmission in the motor cortex. J. Neurochem. 157, 1300–1315. doi: 10.1111/jnc.15214, PMID: 33064315

[ref8] Dyer M. S. Woodhouse A. Blizzard C. A. (2021b). Cytoplasmic human Tdp-43 Mislocalization induces widespread dendritic spine loss in mouse upper motor neurons. Brain Sci. 11. doi: 10.3390/brainsci11070883, PMID: 34209287 PMC8301870

[ref9] Fogarty M. J. Klenowski P. M. Lee J. D. Drieberg-Thompson J. R. Bartlett S. E. Ngo S. T.. (2016a). Cortical synaptic and dendritic spine abnormalities in a presymptomatic Tdp-43 model of amyotrophic lateral sclerosis. Sci. Rep. 6:37968. doi: 10.1038/srep37968, PMID: 27897242 PMC5126629

[ref10] Fogarty M. J. Mu E. W. H. Noakes P. G. Lavidis N. A. Bellingham M. C. (2016b). Marked changes in dendritic structure and spine density precede significant neuronal death in vulnerable cortical pyramidal neuron populations in the Sod1G93A mouse model of amyotrophic lateral sclerosis. Acta Neuropathol. Commun. 4:77. doi: 10.1186/s40478-016-0347-y, PMID: 27488828 PMC4973034

[ref11] Fogarty M. J. Noakes P. G. Bellingham M. C. (2015). Motor cortex layer V pyramidal neurons exhibit dendritic regression, spine loss, and increased synaptic excitation in the presymptomatic hsod1(G93A) mouse model of amyotrophic lateral sclerosis. J. Neurosci. 35, 643–647. doi: 10.1523/JNEUROSCI.3483-14.2015, PMID: 25589758 PMC6605367

[ref12] Handley E. E. Pitman K. A. Dawkins E. Young K. M. Clark R. M. Jiang T. C.. (2017). Synapse dysfunction of layer V pyramidal neurons precedes neurodegeneration in a mouse model of Tdp-43 Proteinopathies. Cereb. Cortex 27, 3630–3647. doi: 10.1093/cercor/bhw185, PMID: 27496536

[ref13] Igaz L. M. Kwong L. K. Lee E. B. Chen-Plotkin A. Swanson E. Unger T.. (2011). Dysregulation of the Als-associated gene Tdp-43 leads to neuronal death and degeneration in mice. J. Clin. Invest. 121, 726–738. doi: 10.1172/JCI44867, PMID: 21206091 PMC3026736

[ref14] Kim J. Hughes E. G. Shetty A. S. Arlotta P. Goff L. A. Bergles D. E.. (2017). Changes in the excitability of neocortical neurons in a mouse model of amyotrophic lateral sclerosis are not specific to corticospinal neurons and are modulated by advancing disease. J. Neurosci. 37, 9037–9053. doi: 10.1523/JNEUROSCI.0811-17.2017, PMID: 28821643 PMC5597984

[ref15] Kuo J. J. Siddique T. Fu R. Heckman C. J. (2005). Increased persistent Na(+) current and its effect on excitability in motoneurones cultured from mutant Sod1 mice. J. Physiol. 563, 843–854. doi: 10.1113/jphysiol.2004.074138, PMID: 15649979 PMC1665614

[ref16] Mackenzie I. R. A. Bigio E. H. Ince P. G. Geser F. Neumann M. Cairns N. J.. (2007). Pathological Tdp-43 distinguishes sporadic amyotrophic lateral sclerosis from amyotrophic lateral sclerosis with Sod1 mutations. Ann. Neurol. 61, 427–434. doi: 10.1002/ana.21147, PMID: 17469116

[ref17] Maekawa S. Leigh P. N. King A. Jones E. Steele J. C. Bodi I.. (2009). Tdp-43 is consistently co-localized with ubiquitinated inclusions in sporadic and Guam amyotrophic lateral sclerosis but not in familial amyotrophic lateral sclerosis with and without Sod1 mutations. Neuropathology 29, 672–683. doi: 10.1111/j.1440-1789.2009.01029.x 19496940

[ref18] Orr B. O. Hauswirth A. G. Celona B. Fetter R. D. Zunino G. Kvon E. Z.. (2020). Presynaptic homeostasis opposes disease progression in mouse models of Als-like degeneration: evidence for homeostatic neuroprotection. Neuron 107, 95–111.e6. doi: 10.1016/j.neuron.2020.04.009 32380032 PMC7529479

[ref19] Pieri M. Albo F. Gaetti C. Spalloni A. Bengtson C. P. Longone P.. (2003). Altered excitability of motor neurons in a transgenic mouse model of familial amyotrophic lateral sclerosis. Neurosci. Lett. 351, 153–156. doi: 10.1016/j.neulet.2003.07.010, PMID: 14623129

[ref20] Pieri M. Carunchio I. Curcio L. Mercuri N. B. Zona C. (2009). Increased persistent sodium current determines cortical hyperexcitability in a genetic model of amyotrophic lateral sclerosis. Exp. Neurol. 215, 368–379. doi: 10.1016/j.expneurol.2008.11.002, PMID: 19071115

[ref21] Reale L. A. Dyer M. S. Perry S. E. Young K. M. Dickson T. C. Woodhouse A.. (2023). Pathologically mislocalised Tdp-43 in upper motor neurons causes a die-forward spread of Als-like pathogenic changes throughout the mouse corticomotor system. Prog. Neurobiol. 226:102449. doi: 10.1016/j.pneurobio.2023.102449, PMID: 37011806

[ref22] Saba L. Viscomi M. T. Caioli S. Pignataro A. Bisicchia E. Pieri M.. (2016). Altered functionality, morphology, and vesicular glutamate transporter expression of cortical motor neurons from a Presymptomatic mouse model of amyotrophic lateral sclerosis. Cereb. Cortex 26, 1512–1528. doi: 10.1093/cercor/bhu317, PMID: 25596588

[ref23] Saba L. Viscomi M. T. Martini A. Caioli S. Mercuri N. B. Guatteo E.. (2019). Modified age-dependent expression of NaV1.6 in an Als model correlates with motor cortex excitability alterations. Neurobiol. Dis. 130:104532. doi: 10.1016/j.nbd.2019.104532 31302244

[ref24] Scekic-Zahirovic J. Sanjuan-Ruiz I. Kan V. Megat S. De Rossi P. Dieterlé S.. (2021). Cytoplasmic Fus triggers early behavioral alterations linked to cortical neuronal hyperactivity and inhibitory synaptic defects. Nat. Commun. 12:3028. doi: 10.1038/s41467-021-23187-9, PMID: 34021132 PMC8140148

[ref25] Schönig K. Weber T. Frömmig A. Wendler L. Pesold B. Djandji D.. (2012). Conditional gene expression systems in the transgenic rat brain. BMC Biol. 10:77. doi: 10.1186/1741-7007-10-77, PMID: 22943311 PMC3520851

[ref26] Turrigiano G. G. (2008). The self-tuning neuron: synaptic scaling of excitatory synapses. Cells 135, 422–435. doi: 10.1016/j.cell.2008.10.008 PMC2834419 18984155

[ref27] Turrigiano G. (2012). Homeostatic synaptic plasticity: local and global mechanisms for stabilizing neuronal function. Cold Spring Harb. Perspect. Biol. 4:a005736. doi: 10.1101/cshperspect.a005736 22086977 PMC3249629

[ref28] Vallano M. L. Beaman-Hall C. M. Mathur A. Chen Q. (2000). Astrocytes express specific variants of CaM Kii delta and gamma, but not alpha and beta, that determine their cellular localizations. Glia 30, 154–164. doi: 10.1002/(SICI)1098-1136(200004)30:2<154::AID-GLIA5>3.0.CO;2-S 10719357

[ref29] Veres J. M. Andrasi T. Nagy-Pal P. Hajos N. (2023). Camkiiα promoter-controlled circuit manipulations target both pyramidal cells and inhibitory interneurons in cortical networks. eNeuro 10, ENEURO.0070–ENEU23.2023. doi: 10.1523/ENEURO.0070-23.2023, PMID: 36963833 PMC10088982

[ref30] Vucic S. Cheah B. C. Yiannikas C. Kiernan M. C. (2011). Cortical excitability distinguishes Als from mimic disorders. Clin. Neurophysiol. 122, 1860–1866. doi: 10.1016/j.clinph.2010.12.062, PMID: 21382747

[ref31] Vucic S. Kiernan M. C. (2008). Cortical excitability testing distinguishes Kennedy's disease from amyotrophic lateral sclerosis. Clin. Neurophysiol. 119, 1088–1096. doi: 10.1016/j.clinph.2008.01.011 18313980

[ref32] Vucic S. Nicholson G. A. Kiernan M. C. (2008). Cortical hyperexcitability may precede the onset of familial amyotrophic lateral sclerosis. Brain 131, 1540–1550. doi: 10.1093/brain/awn071 18469020

[ref33] Vucic S. Pavey N. Haidar M. Turner B. J. Kiernan M. C. (2021). Cortical hyperexcitability: diagnostic and pathogenic biomarker of Als. Neurosci. Lett. 759:136039. doi: 10.1016/j.neulet.2021.136039, PMID: 34118310

[ref34] Wainger B. J. Kiskinis E. Mellin C. Wiskow O. Han S. S. Sandoe J.. (2014). Intrinsic membrane hyperexcitability of amyotrophic lateral sclerosis patient-derived motor neurons. Cell Rep. 7, 1–11. doi: 10.1016/j.celrep.2014.03.019, PMID: 24703839 PMC4023477

[ref35] Zhang W. Zhang L. Liang B. Schroeder D. Zhang Z. W. Cox G. A.. (2016). Hyperactive somatostatin interneurons contribute to excitotoxicity in neurodegenerative disorders. Nat. Neurosci. 19, 557–559. doi: 10.1038/nn.4257, PMID: 26900927 PMC4811704

