# Supplementary figures and images for: Synaptic remodeling follows upper motor neuron hyperexcitability in a rodent model of TDP-43
Source: Front Cell Neurosci. 2023 Oct 24;17:1274979. doi: 10.3389/fncel.2023.1274979 (PMC10628445; doi:10.3389/fncel.2023.1274979)

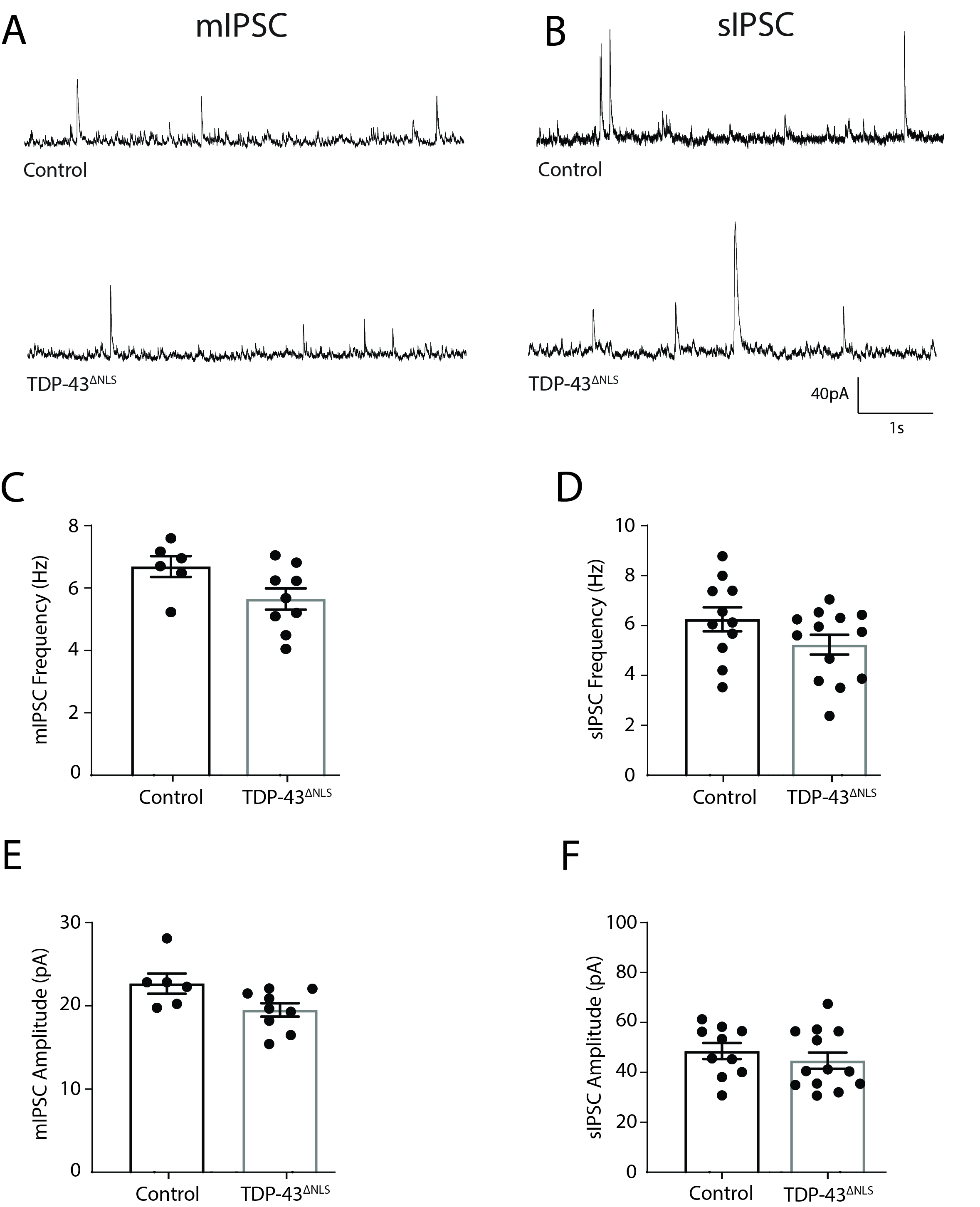

Supplement: Supplementary file 1 [file Image_1.JPEG]
